# Safe wrapping of the borderline dilated ascending aorta during aortic valve replacement

**DOI:** 10.1186/1749-8090-2-15

**Published:** 2007-02-22

**Authors:** Ernesto Tappainer, Vinicio Fiorani, Andrea Nocchi, Ermal Likaj, Sabjan Memishaj, Mario Zogno

**Affiliations:** 1 Cardiac Surgery Unit, "Carlo Poma" Hospital, Viale Albertoni 1, 46100 Mantua, Italy; 2 Cardiovascular Department, University Hospital Center "Mother Teresa", Rruga e Dibres 372, Tirana, Albania

## Abstract

**Background:**

Techniques of reduction aortoplasty are widely published in the literature with conflicting results. External support seems to be an important factor in preventing recurrence but, in some cases, this technique caused erosion of the aorta because of the wrinkles the prosthesis creates in the rear side of the aorta.

**Case presentation:**

A 73 year old patient with aortic valve stenosis and borderline dilated ascending aorta had aortic valve replacement and simple wrapping without aortoplasty. To avoid the formation of wrinkles, the dacron external support was tailored appropriately to obtain a curved, custom-made prosthesis. This custom-made prosthesis had the same diameter as the dilated aorta and, after valve replacement, fitted it properly. After 18 months neither computerized axial tomography nor ecocardiography detected wrinkles or dilatation recurrence.

**Conclusion:**

A safe, simple and probably new way to prepare an external wrapping is presented, which in this patient respected the shape of the aorta and prevented the formation of wrinkles in the prosthesis and possible complications such as wall erosion.

## Background

Replacement of the aorta with a vascular dacron prosthesis is a frequently performed procedure for thoracic aortic aneurysms [1]. The anatomo-pathology of aneurysms involving the ascending aorta is variable. This variability has led to a number of different operations and techniques for the repair of the aneurysm and associated pathologies, especially aortic valve diseases. Radical treatment like Bentall, modified Bentall, or Cabrol procedures, are mandatory in case of enlargement of the ascending aorta including sinuses of Valsalva with displacement of coronary arteries and aortic valve insufficiency. On the other hand, conservative, less radical and simple operations could be performed in cases where borderline dilatation of the ascending aorta is encountered during valve replacement for aortic stenosis. Aortoplasty is a well known conservative method, which was first described by Robicsek [2]. This technique consists in resection of a longitudinal oval segment of the ascending aortic wall followed by reinforcement by a dacron vascular prosthesis, which is wrapped around. In selected cases with borderline dilated aorta, rather than doing nothing, we propose an even less invasive procedure we used in a patient with good results.

## Case presentation

**Figure 1:**
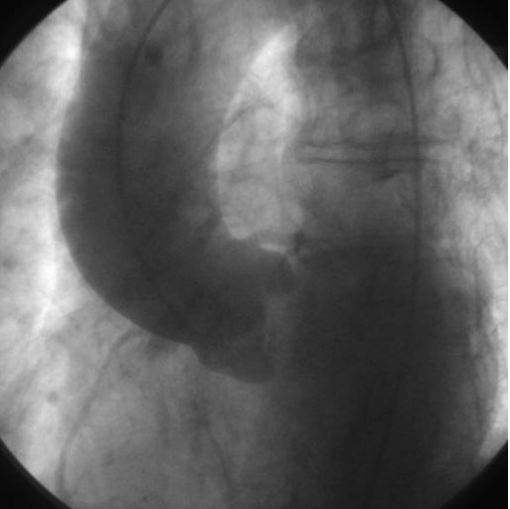
preoperative aortography.

A 73 years old male patient was found to have calcified aortic stenosis after a syncopal episode. He had a history of hypertension and a previous diagnosis of obstructive chronic broncopneumopathy. He was 170 cm tall, his weight was 75 kilos and body surface area was 1.85 sqm. At preoperative investigations he was found to have a borderline dilated ascending aorta of about 45 mm (Figure 1). At the time of aortic valve replacement a written informed consent was obtained even for treatment of the ascending aorta if needed but no specifications were made about the type of intervention.

### Operative Technique

**Figure 2:**
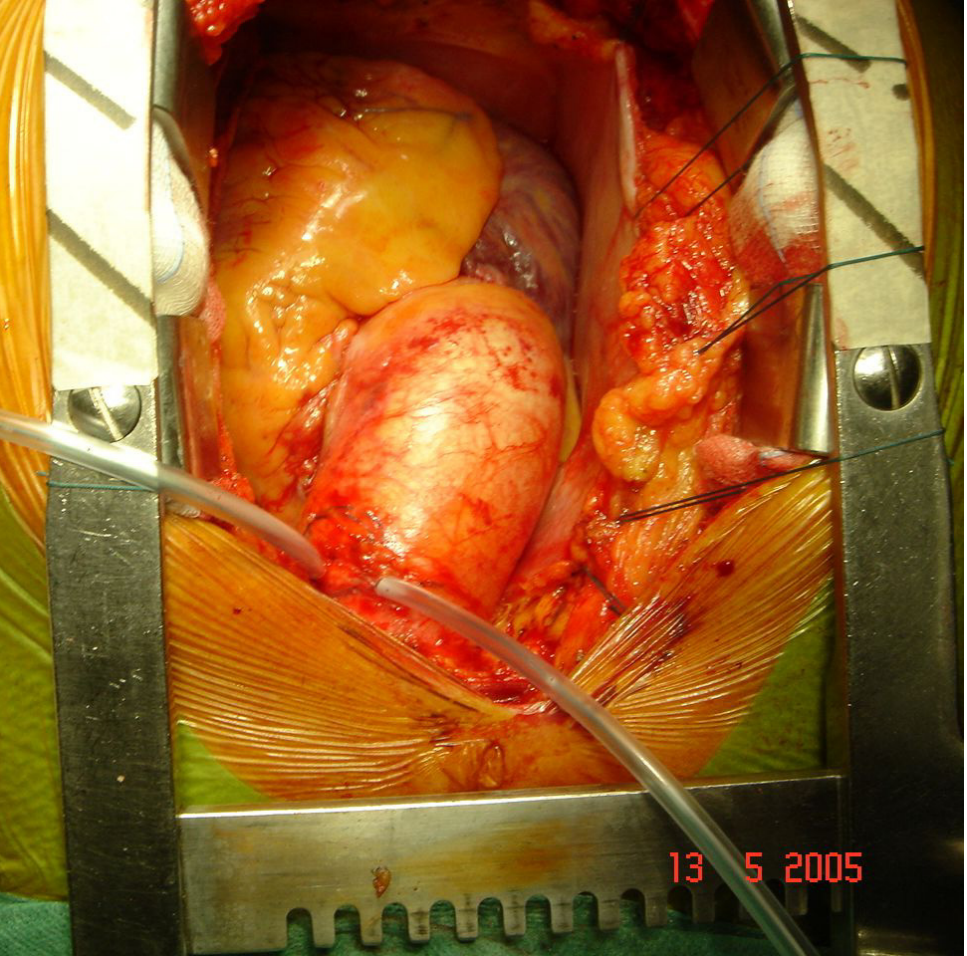
intraoperative image before cross-clamping.

**Figure 3:**
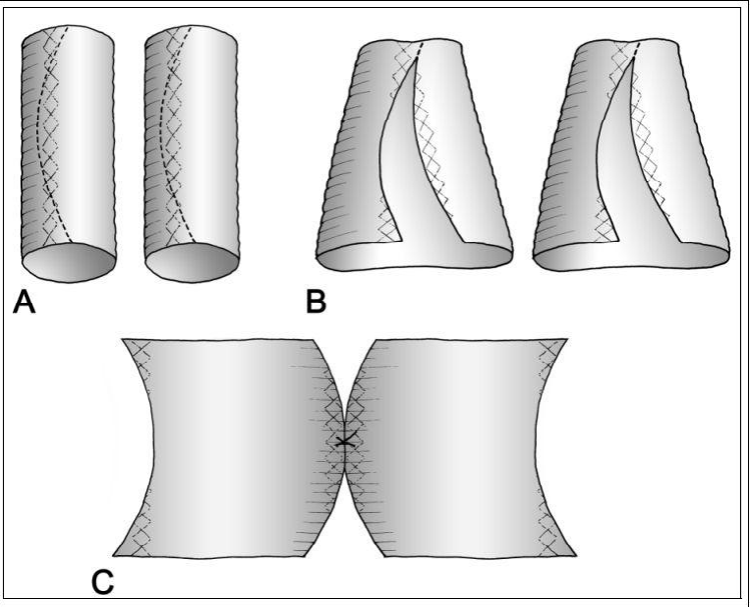
drawing of custom-made prosthesis preparation.

**Figure 4:**
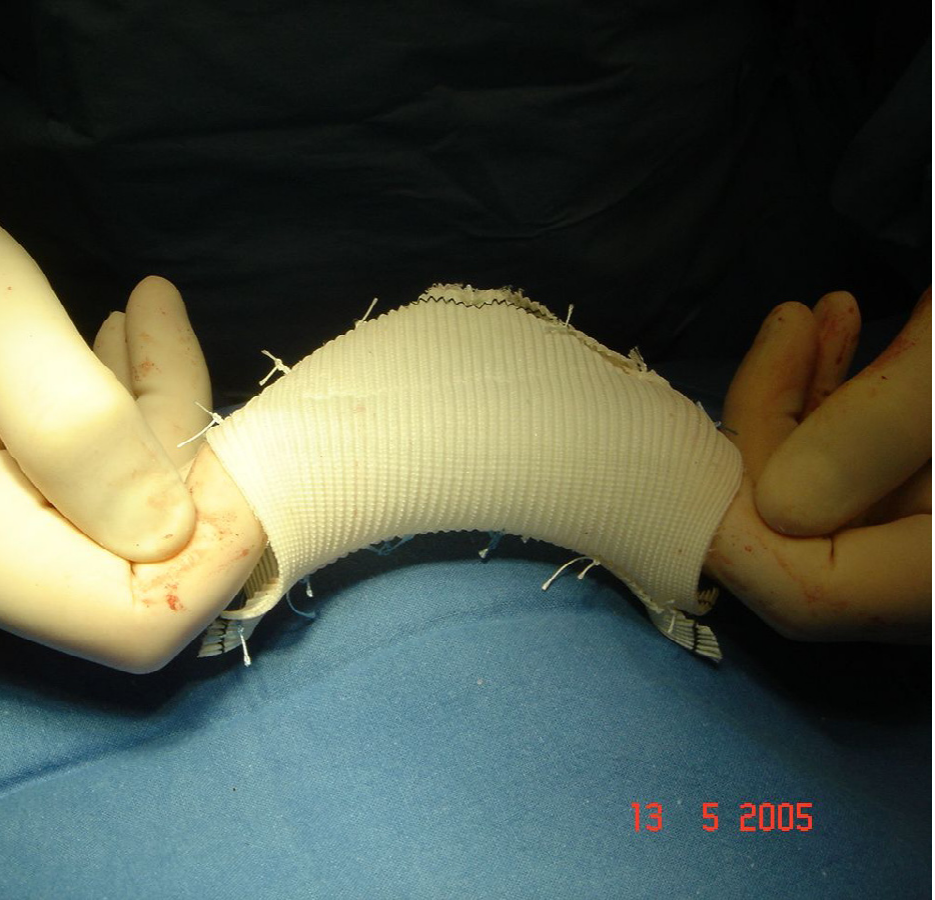
custom-made prosthesis before insertion.

**Figure 5:**
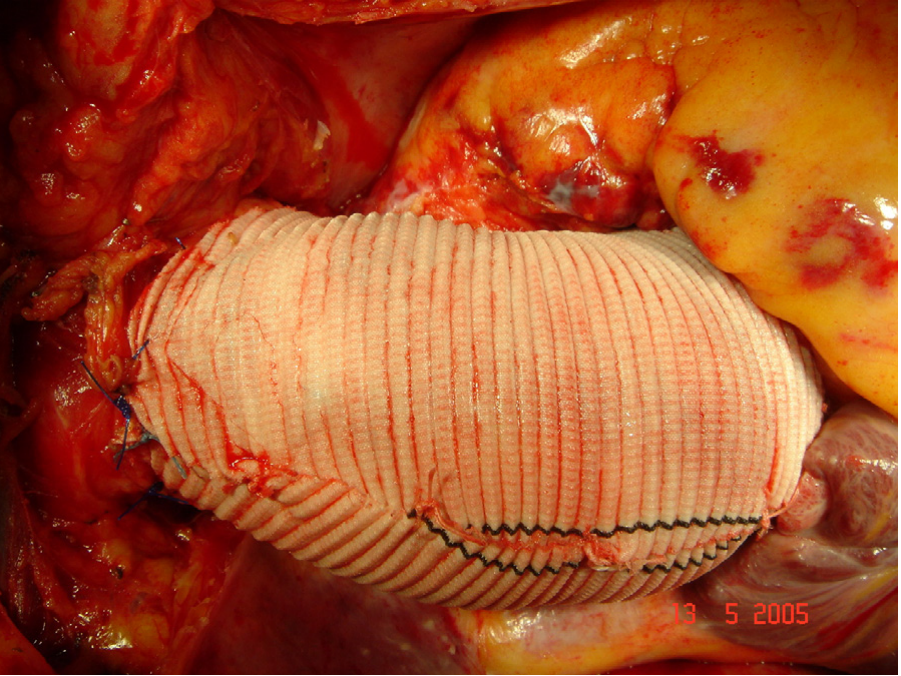
**intraperative image before patient closure**. The custom-made prosthesis for wrapping appears to fit the ascending aorta perfectly without wrinkles or angulations. Only few precautionary adventitial stitches were used.

After median sternotomy, cannulations of distal ascending aorta, right atrio-caval, coronary sinus and left ventricle via right superior pulmonary vein were performed. For myocardial protection intermittent anterograde and retrograde warm blood cardioplegia was used. At inspection, the mid ascending aorta showed an external diameter of about 50 mm (Figure 2). Aortic wrap was constructed before aortic cross-clamping: a vascular dacron prosthesis 12 × 26 mm (Woven Dacron Gelweave, Vascutek Ltd, Inchinnan, Scotland, UK) was taken. The prosthesis was cut into two halves of 6 cm length (Figure 3a). Both halves were opened longitudinally by a curved cut. As a reference the black lines on the prostheses were used. The cuts were about 4 mm apart from the black line in the centre of the length of the prostheses, and 4 mm apart from the black line on the opposite sides at the extremities (Figure 3b). Thus two dacron sheets were obtained from the prostheses, each of them having one concave and one convex side. Finally the sheets were joined by suturing the two convex sides together and the two concave sides together too (Figure 3c). A curved dacron hose 5 cm in diameter was obtained for external wrapping of the ascending aorta (Figure 4). The joining sutures of the two sheets were made of single separate stitches so that the extremities could be shortened if needed. After cross clamping, the aorta was cut transversally above the commissures at the sinotubular junction. Aortic valve replacement was then performed as usual. A 25-mm biological prosthetic aortic valve was inserted with 15 pledget-supported stitches. Then, before aortic closure, the posterior aspect of the ascending aorta was freed completely from the pericardial reflection up to the innominate artery. In this way the custom-built prosthesis was easily inserted, like a trouser leg, to wrap the ascending aorta. The aorta was closed by a continous running suture. The cross clamp was released after 64 min while extracorporeal circulation was arrested after 78 min without inotropes. Then, the prosthesis was pulled down to cover the suture line and fixed with few adventitial stitches. No solid transmural stitches were needed because the curved prosthesis fitted the curved ascending aorta best. Similarly, after decannulation, the prosthesis was pulled up to cover the cannulation site. Therefore the whole ascending aorta was covered by the prosthesis which appeared to fit perfectly without wrinkles or bends (Figure 5).

## Results

**Figure 6:**
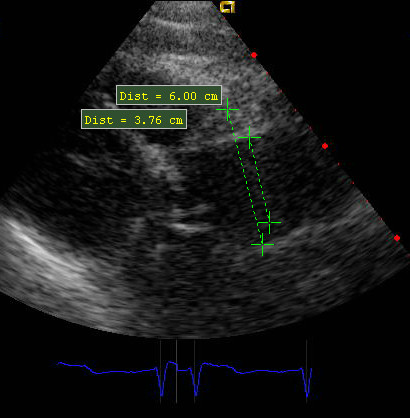
**Trans-thoracic ECO 18 months later**. Wrapping does not tighten the aortic root which does not reach the diameter of 50 mm of the custom-made prosthesis.

**Figure 7:**
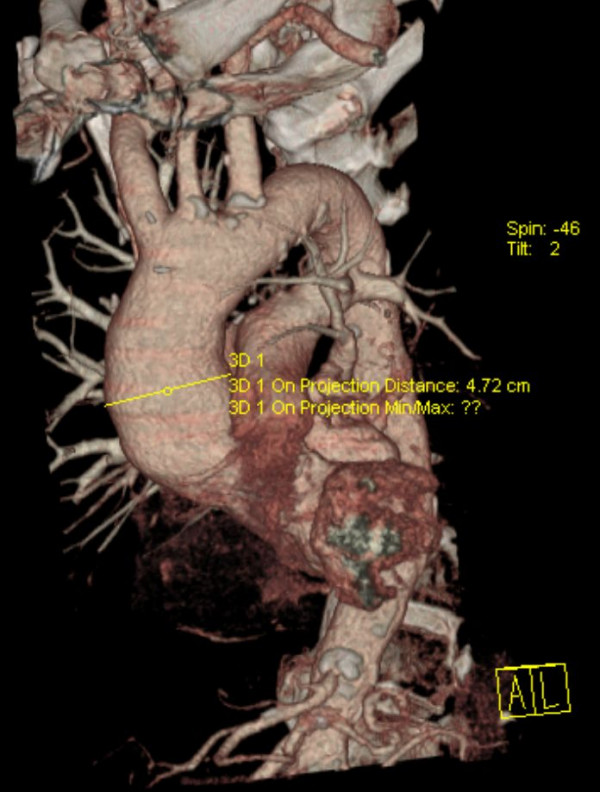
**TAC 18 months later**. No wrinkles are detectable nor further dilatation.

The patient did well. At the follow up 18 months later, the patient was doing well. Transthoracic echocardiography (Figure 6) and computed axial tomography (Figure 7) revealed no further aortic dilatation nor wrinkles or bends of the wrapping.

## Discussion

The case we present has three characteristics which induced us to apply the technique: aortic valve replacement, old age and borderline dilated aorta. The dacron wrap may provide reinforcement for the weakened wall of the ascending aorta. Use of aortoplasty with external wrapping, rather than replacement, in case of moderately sized ascending aneurysm of the aorta is still controversial. A series of patients operated on using different techniques are widely published in the literature, and the results are conflicting [3,4]. The tendency to dilate is related to the underlying intrinsic wall deficiency [5]. Reduction aortoplasty eliminates the aneurysm but it does not prevent recurrence without any external support. Aortoplasty with external wrapping reduces wall stress by restoring both normal aortic diameter and wall strength [5]. On the other hand, wrapping was found to be unnecessary in cases of aortic stenosis because no dilatation recurrence was found in a group of patients after unsupported aortoplasty [6]. Moreover, wrapping could cause erosion in the rear side of the aorta by the wrinkles of the prosthesis itself, due to the fact that the aorta is curved while the prosthesis is straight [7]. Nevertheless, we sometimes have to deal with old patients with a borderline dilatation of the ascending aorta and aortic valve stenosis. We do not know if dilatation is related to the intrinsic wall weakness rather than to the hemodynamic disturbance, while old age would suggest the least possible aggressive treatment. As an alternative to doing nothing we propose a simple external wrapping, without reduction aortoplasty, which acts as a good measure to prevent further dilatation of the aorta. With the increasing mean age of patients in recent years, there could be an increasing need for a lower risk procedure. Indeed wrapping is considered a good compromise in older patients with a borderline dilated aorta, especially during operations for other cardiac pathology [1,3]. A lower postoperative complication rate was observed with this technique, particularly regarding perioperative myocardial infarction, cerebro-vascular complications, and reexploration for haemostasis [3]. While the replacement of the aorta appears to be an over-treatment of the disease, our technique has the advantage of simplicity, it preserves the endothelial lining of the ascending aorta which is lost with prosthetic substitution, it avoids manipulation of the coronary ostia, it does not need anticoagulation therapy and it can be performed with the same cross-clamp and bypass-time required for uncomplicated AVR. Moreover, during ascending aorta replacement an open distal anastomosis has often to be performed. This requires a hypothermic circulatory arrest with all its consequences (i.e. lengthy operation, bleeding, metabolic imbalance, neurologic injury, etc.).

There are two technical problems during the wrap preparation. First no prosthesis is ready for use because the maximum available graft diameter is 34 mm, which is insufficient to fit an ascending aorta of 50 mm. This is the limit beyond which the ascending aorta has to be replaced by a vascular prosthesis [1,3]. Thus no graft is made especially to reinforce the moderately dilated ascending aorta. Secondly, we have to fit a straight prosthesis to a curved aorta. Vascular prostheses have a crimped fabric, which allows the prostheses to be moderately bended without angulations. However, to wrap an aorta of 50 mm in diameter using one of them, you have to open and use it in the opposite direction, i.e. lengthwise rather than widthwise, so that the crimping fabric becomes ineffective. This is not a problem in a straight artery, but being the ascending aorta somewhat curvilinear, the prosthesis develops wrinkles in aortic concavity. Wrinkles of the prosthesis are responsible for erosion of the aortic wall [7]. Thus aortic wrapping could became more dangerous than dilatation itself. Histopathologic modifications of the aortic tissues in the area covered by external prosthetic wrapping have been described by Neri et al. [8]. They found extensive aortic wall degeneration and supposed that interruption of vasa vasorum, chronic foreign-body reaction, and constriction of the aortic wall layers between opposite forces (external banding and aortic pressure) may interfere with the tissue metabolism [8]. Moreover, like Bauer et al. [7], they found that the lesions of the aortic wall were in posterior aspect of the ascending aorta, that is its concavity, where wrinkles are likely to develop [7]. We hope that our custom-made prosthesis, which respects the aortic curvature and the crimping direction of the prostethic tissue, could minimize wall degeneration.

On the basis of this experience we are ready to perform such procedure in other patients if it is necessary.

## Conclusion

We have presented a safe, simple and probably new technique for external wrapping of the borderline dilated ascending aorta - during aortic valve replacement - by a manually preprepared prosthesis which respects the shape of the aorta. It also prevents wrinkles of the prosthesis and ensuing complications.

## Competing interests

The author(s) declare that they have no competing interests.

## Authors' contributions

ET and VF invented the technique and performed the operation. MZ made a critical analysis and approved the technique. AN took care of the patients in the ward. EL and SM attended to the bibliography and the follow up.

All authors read and approved the final manuscript
